# Efficient approaches for large-scale GWAS with genotype uncertainty

**DOI:** 10.1093/g3journal/jkab385

**Published:** 2021-12-04

**Authors:** Emil Jørsboe, Anders Albrechtsen

**Affiliations:** 1 Department of Biology, The Bioinformatics Centre, University of Copenhagen, 2200 Copenhagen N, Denmark; 2 Faculty of Health and Medical Sciences, Novo Nordisk Foundation Center for Basic Metabolic Research, University of Copenhagen, 2200 Copenhagen N, Denmark

**Keywords:** admixture, association mapping, case-control study, next-generation sequencing, quantitative traits

## Abstract

Association studies using genetic data from SNP-chip-based imputation or low-depth sequencing data provide a cost-efficient design for large-scale association studies. We explore methods for performing association studies applicable to such genetic data and investigate how using different priors when estimating genotype probabilities affects the association results. Our proposed method, ANGSD-asso’s latent model, models the unobserved genotype as a latent variable in a generalized linear model framework. The software is implemented in C/C++ and can be run multi-threaded. ANGSD-asso is based on genotype probabilities, which can be estimated using either the sample allele frequency or the individual allele frequencies as a prior. We explore through simulations how genotype probability-based methods compare with using genetic dosages. Our simulations show that in a structured population using the individual allele frequency prior has better power than the sample allele frequency. In scenarios with sequencing depth and phenotype correlation ANGSD-asso’s latent model has higher statistical power and less bias than using dosages. Adding additional covariates to the linear model of ANGSD-asso’s latent model has higher statistical power and less bias than other methods that accommodate genotype uncertainty, while also being much faster. This is shown with imputed data from UK Biobank and simulations.

## Introduction

Genome-wide association studies (GWASs) have classically been done to study genotype–phenotype associations. However, a slightly different design of GWAS is using low depth next-generation sequencing (NGS) data, because in such cases the genotype cannot be inferred accurately. Low depth sequencing provides a cost-efficient design, where the number of individuals studied can be increased many folds compared with high depth sequencing, since each individual will be a lot cheaper to sequence. The statistical power to detect associations increases with the number of individuals while only dropping slightly with lower depth and therefore this design provides good statistical power to detect associations (Pasaniuc *et al.* 2012). Another approach that is commonly used in GWAS is performing haplotype imputation from SNP-chips to infer missing genotypes which also generates genetic data with uncertainty on the inferred genotype.

A recent successful GWAS (Liu et al. 2018) with low depth NGS data has shown the viability of this approach. In Liu et al. (2018), around 140,000 individuals had a noninvasive prenatal test for fetal trisomy with low depth sequencing with an average sequencing depth of 0.1X. Haplotype imputation was performed on the low depth NGS data. For the association testing a score test approach using a linear model framework (Skotte et al. 2012) implemented in ANGSD (Korneliussen et al. 2014) was used, as this method takes genotype uncertainty into account. Despite the low sequencing depth several novel associations were discovered. This provides an example of a study where using methods that account for the genotype uncertainty in low depth NGS data, provides good statistical power for detecting associations.

In association studies, genotype uncertainty can be taken into account using a latent variable model that sums over the possible genotype states. Using latent variable models can have advantages compared with calling genotypes for low depth NGS data (Skotte et al. 2012). Skotte et al. (2012) implemented a score test where the coefficients are not estimated under the alternative hypothesis making the method computationally very fast; however, this means the effect size of the genotype is not estimated. In this article, we will introduce ANGSD-asso’s latent model, it works in a generalized linear model (GLM) framework; it estimates the effect size of the unobserved genotype. This method can in practice be run almost as fast as the score test. ANGSD-asso’s latent model uses a maximum likelihood approach; more specifically, we will make use of the EM algorithm to maximize the likelihood, treating the unobserved genotype *G* as a latent variable. Using a GLM framework enables us to include covariates thereby adjusting for possible confounders, such as population structure. We have implemented an EM algorithm that converges fast and that can be run multi-threaded, making the analysis of large data sets possible. We have also implemented a hybrid model in ANGSD-asso combining the speed of the score test with the desired properties of the EM algorithm-based approach. Using the EM algorithm for doing maximum likelihood estimation in a GLM framework using genotype probabilities has been implemented in SNPTEST (Marchini *et al.* 2007). However, SNPTEST cannot jointly estimate effect sizes of genotype and covariates and is not computationally efficient. We have designed a faster implementation that allows for the association analysis of large-scale NGS data sets.

A common practice for performing association analysis based on genotype probabilities is using genetic dosages, which are the expected genotypes calculated from the genotype probabilities. Dosages are easy to implement into most existing methods as the genotype can be directly replaced by the dosage. However, dosages do not convey the uncertainty on the genotype as fully as genotype likelihoods or genotype probabilities. In Zheng et al. (2011), they show a gain in power when using genotype probability-based methods compared with dosages, but only for small studies with variants with large effect sizes. However, they did not look at how sequencing depth and phenotype correlation might affect this. This could happen in a case-control study, if cases and controls were sequenced at different places, or if the total data set is merged from other smaller heterogeneous data sets, creating a systematic difference in the sequencing depth. We investigate this sequencing depth bias through simulations of a large-scale association study and evaluate the performance of our genotype probability-based method compared with using dosages. In order to be able to assess how a sequencing depth bias might impact the analyses, we focus on simulating genetic data as low depth NGS data. However, we show that a scenario similar to this could also happen with data based on haplotype imputation.

ANGSD-asso, and the score test and SNPTEST all work on genotype probabilities (also known as the “posterior probability”). The genotype probabilities can be calculated from the genotype likelihoods, which in turn can be calculated from the NGS data (Nielsen *et al.* 2011). For more on genotype likelihoods and NGS data see Supplementary Section S5.2.

Another aspect, we will explore is how to estimate genotype probabilities taking population structure into account, when performing association studies with low depth sequencing data. Population structure is a common confounder in association studies that need to be addressed properly (Cardon and Palmer 2003; Freedman *et al.* 2004; Marchini *et al.* 2004). For low depth sequencing data, the genotype probabilities are often obtained by using a prior based on the allele frequency estimated from the same sequencing data. We will refer to this allele frequency as the “sample allele frequency”. Using this prior assumes a homogeneous population without population structure. We therefore propose to use a different prior when dealing with structured populations based on individual allele frequencies. The individual allele frequency is the weighted average population frequency where the admixture proportions for each individual are the weights. The individual allele frequency for a site has to be calculated for each individual, as their admixture proportions might differ. This takes both the frequency of the variant and the ancestry of every individual into account. We therefore want to investigate how this approach compares to using the sample allele frequency prior in different scenarios. We will look at this both with regard to statistical power the false positive rate.

## Methods

**Figure 1:**
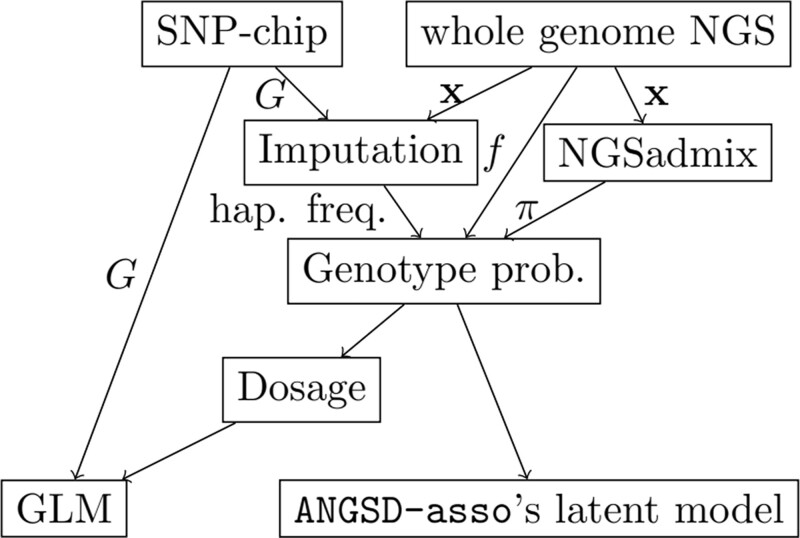
Schematic of workflow for performing association studies with genetic data. **x** is the sequence data that can be converted to genotype likelihoods, *G* is the genotypes. *π* is the individual allele frequencies and *f* is the sample allele frequency both of which can be used as priors when estimating the genotype probabilities. Data either gets generated using SNP-chips or doing NGS. The NGS data can be converted into genotype probabilities assuming no population structure using the sample allele frequency, or assuming population structure and then using principal component analysis (PCA) to generate genotype probabilities. SNP-chip genotypes can be analyzed directly. Both kinds of data can be imputed using haplotype frequencies for generating genotype probabilities. The genotype probabilities can be analyzed with ANGSD-asso’s latent model or converted to dosages and then be analyzed with a GLM.

NGS produces short reads that are mapped to a reference genome. From the aligned reads the probability of observing these reads at a given site for a certain genotype can be calculated. This is known as the genotype likelihood (Nielsen *et al.* 2011). For more on the genotype likelihood and how to calculate it see the Supplementary Section S5.2. The genotype likelihoods together with a genotype prior can be used to calculate the probability of the genotype given the data which is referred to as the genotype probability. For an overview of the relationship between the different kinds of genetic data, and how they can be processed and analyzed in association studies see Figure 1.

### ANGSD-asso’s latent model

We model the data using a maximum likelihood approach in a GLM framework. This enables us to test for an association without observing the genotype *G* directly. Rather we observe our NGS data (**x**) from which we can calculate the genotype probability p(G|x). We write the likelihood for our phenotype data (**y**) given our sequencing data (**x**) and covariates (*Z*):

(1) $$p(y|x,Z)=\prod_{i=1}^{N}p(y_{i}|x_{i},z_{i})=\prod_{i=1}^{N}\sum_{g\in \{0,1,2\}}p(y_{i}|G_{i}=g,z_{i})p(G_{i}=g|x_{i}),$$

where we use the law of total probabilities to introduce the genotype as a latent variable *G*. *N* is the number of individuals, $y=(y_{1},y_{2},\ldots ,y_{N})$ is a vector of our observed phenotype, $x=(x_{1},x_{2},\ldots ,x_{N})$ is a vector of sequencing data and $Z=(z_{1},z_{2},\ldots ,z_{N})$ is a *N *×* c* matrix with the additional covariates. We see that the trait *y_i_* is conditionally independent of the sequencing data given the genotype [meaning $p(y_{i}|G_{i}=g,x_{i},z_{i})=p(y_{i}|G_{i}=g,z_{i})$]. We can calculate the genotype probability from the sequence data, for example, by using the sample allele frequency as a prior, by assuming that the genotype is conditionally independent of the covariates, given the sequencing data and the frequency *f* (meaning $p(G_{i}=g|x_{i},z_{i},f)=p(G_{i}=g|x_{i},f)$); however, for simplicity we omit *f* from the likelihood.

This allows us to write the likelihood, also introducing the parameters of our GLM θ=(α,β,γ), again we assume that the genotype is conditionally independent of the covariates given the sequencing data:

(2) $$L(\theta )\propto p(y|x,Z,\theta )=\prod_{i=1}^{N}p(y_{i}|x_{i},z_{i},\theta )$$

(3) $$=\prod_{i=1}^{N}\sum_{g\in \{0,1,2\}}p(y_{i}|G_{i}=g,z_{i},\theta )p(G_{i}=g|x_{i}).$$

And the log-likelood then becomes

(4) $$=\sum_{i=1}^{N}\;\text{log}\;\left(\sum_{g\in \{0,1,2\}}p(y_{i}|G_{i}=g,z_{i},\theta )p(G_{i}=g|x_{i})\right).$$

Assuming the term $p(y_{i}|G_{i}=g,z_{i},\theta )$ follows a normal distribution, given the genotype *G* takes the value *g*, the covariates *Z* and the linear coefficients *θ*, the mean is given by:

(5) $$\eta_{i}=\alpha +\beta G_{i}+\sum_{c}\gamma_{c}z_{ic}+\epsilon_{i}.$$

Equation (4) is the log-likelihood function that we want to maximize with regard to the parameters *θ*. For maximization, we use the EM algorithm where our latent variable is the unobserved genotype *G*. This is done by weighted least squares regression, where the parameters *θ* are estimated. For the full derivations of this see the Supplementary Sections S7 and S7.1. We have also implemented logistic and Poisson regression where we have introduced a link function for *η_i_* for Equation (5) and changed the distribution for $p(y_{i}|G,z_{i},\theta )$ accordingly. For more information on this see the Supplementary Section S6.

Furthermore, standard errors on the estimated effect sizes are estimated using the observed Fisher information matrix as in Lake *et al.* (2003) and Skotte et al. (2019). In order to achieve faster convergence of the latent model, we first do regression on the genotype dosages. We then use the coefficients obtained from the dosage regression as the starting guess for the coefficients for the EM algorithm (we refer to this as priming).

#### Implementation of ANGSD-asso

The three methods in ANGSD-asso for association analysis are implemented in the ANGSD framework (Korneliussen et al. 2014), allowing multi-threaded analysis. ANGSD can be downloaded from its github page: https://github.com/ANGSD/angsd. The latent variable model is -doAsso 4, the hybrid model (see Supplementary Methods) is -doAsso 5 and the dosage model (see Supplementary Methods) is -doAsso 6. ANGSD-asso works on genotype probabilities in the BEAGLE, BGEN, and BCF/VCF file formats or directly from BAM files and the other file formats allowed in ANGSD.

### Individual allele frequency prior

When estimating the genotype probabilities for low depth sequencing data, it is important to have an appropriate prior, when dealing with genotype data in a structured population. The sample frequency *f* of an allele might not describe the occurrence of an allele across individuals very well. This is due to the fact that the frequency of an allele might differ drastically between different ancestries. Therefore a prior based on the sample frequency will not work well in a structured population. If we have a discrete number of ancestral populations then by using a weighted average of the ancestral frequencies, we can calculate the individual allele frequency (*π_ij_*), for individual *i* for site *j*, across *k* populations:

(6) $$\pi_{ij}=\sum_{k}q_{ki}f_{jk}.$$

where *f_jk_* is the frequency of the *j*th site in population *k* and *q_ik_* is the admixture proportion of population *k* for individual *i*. In order to estimate the individual allele frequencies, we will have to first estimate the ancestral frequencies and the admixture proportions. For NGS data this can be done using NGSadmix (Skotte et al. 2013) and for genotypes, this can be done using ADMIXTURE (Alexander et al. 2009). We use the approach from NGSadmix when inferring population frequencies, in our simulations (see Supplementary Methods for more) with low depth sequencing data in a structured population, assuming admixture proportions are known.

Another approach is (Hao et al. 2015) or PCAngsd (Meisner and Albrechtsen 2018), where the population structure between individuals is modeled using principal components rather than a discrete number of ancestral populations. When the individual allele frequencies have been generated, we can calculate more accurate genotype probabilities, this can be done using Bayes’ formula as laid out in Equation (3) (Supplementary Methods; where we replace *f* by *π*). *p*(*G*) can be calculated using our individual allele frequency assuming Hardy–Weinberg proportions:

(7) $$p(G_{ij}|\pi_{ij})=\{\begin{array}{cc} {\left(1-\pi_{ij}\right)}^{2}\; & G_{ij}=0\\ 2\pi_{ij}(1-\pi_{ij})\; & G_{ij}=1\\ {\left(\pi_{ij}\right)}^{2}\; & G_{ij}=2. \end{array}$$

#### Simulation of phenotypes

We simulate the phenotypes under a standard additive model with a normally distributed phenotype. The mean given is given by effect *β* from the genotype (*g*) and the ancestry/admixture proportions (*q*) with effect *γ* from being population 1 and SD 1, according to Equation (5). The mean for each simulation can also be seen in the last column of Table 1. We simulate different scenarios respectively with and without effect of the genotype, ancestry or sequencing depth, in order to be able to assess how each of them affects the result. Specifically, we wanted to explore scenarios where ancestry had a strong effect and we therefore chose *γ* to have a value of 1. Furthermore, for the genotype effect size *β*, we chose a range of values that we deemed realistic. Finally, we also wanted to explore the impact of correlation between sequencing depth and the phenotype, and how to handle this. We therefore introduced sequencing depth and phenotype correlation in our simulations. Essentially, this means that having a large phenotype value or being a case will make it more likely that an individual is in the high depth group. This is described in more detail in Supplementary Sections S1.3 and S4.

**Table 1:** Overview of simulations

Scenario	Allele frequency	*N*	Population structure	Sequencing depth and phenotype	Simulated phenotype mean
1	0.45	1,000	No	Correlated	0
2	0.45	1,000	No	Not correlated	β·g
3	(0.9,0.1)	1,000	Yes	Correlated	q·γ
4	(0.9,0.1)	1,000	Yes	Not correlated	β·g+q·γ
5	0.45	1,000	No	Correlated	β·g
6	(0.9,0.1)	1,000	Yes	Correlated	β·g+q·γ

We simulate under a standard additive model with a normally distributed phenotype with a mean given in the last column, and SD 1, according to Equation (5). *N* is the number of individuals, *g* is the genotype with effect size *β* and *q* is the ancestry proportion with effect size*γ*. In scenarios 1 and 3, there is no effect of the genotype. In scenarios 3, 4, and 6, there is population structure with two ancestral populations with allele frequency of 0.9 and 0.1. The sequencing depth and phenotype correlation are simulated using (Equation 5;Supplementary Methods).

## Results and discussion

We wanted to assess which prior performs best for generating the genotype probabilities when performing association studies with low depth sequencing data. We simulate different scenarios with and without population structure and with and without sequencing depth and phenotype correlation. For each of these scenarios, we both applied a sample allele frequency prior and an individual allele frequency prior and evaluated their performance with regard to false positive rate and statistical power to detect an association. Genotype dosages or the expected genotypes are often used in association studies, in order to try and account for the uncertainty on the genotype. However, dosages can be uninformative especially with low depth sequencing data. We did simulations in order to investigate the statistical power to detect an association, when we model the full genotype probabilities instead of using just the genotype dosage. We use ANGSD-asso latent model and dosage model for this. Last, we compared ANGSD-asso latent model with the similar model in SNPTEST in terms of bias, statistical power and computational speed. An overview of the simulation scenarios is shown in Table 1.

### Evaluation of using different priors

#### Using different priors in a homogeneous population

In scenario 1, we simulate data without genotype effect, and population structure but with sequencing depth and phenotype correlation. We observe no inflation of the false positive rate for both priors (Supplementary Figure S1). This is expected since these priors become identical in the absence of population structure. In scenario 2, we add a genotype effect and again we observe no difference in the two priors in terms of statistical power (Supplementary Figure S2).

#### Using different priors in a structured population

**Figure 2:**
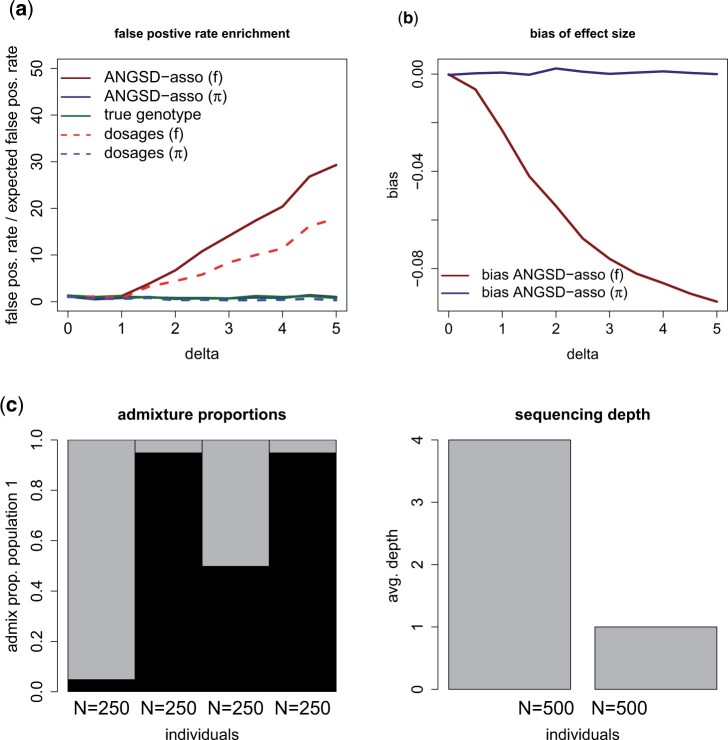
Simulation scenario 3 without genotype effect but with population structure and with varying degree of sequencing depth and phenotype correlation (*δ*). There is an effect of ancestry of population 1 (*γ *= 1) which is adjusted for in the linear model. We use a significance threshold of $10^{-3}$. Each point is based on 10,000 simulations each of 1000 individuals. (**A**) We show the false positive rate divided by the expected false positive rate ($10^{-3}$), using ANGSD-asso’s latent model and dosage model respectively with a sample frequency prior (f) and an individual allele frequency prior (*π*). (**B**) Bias of the estimated effect sizes. (**C**) The simulated admixture proportions and the mean sequencing depth for the simulated individuals.

**Figure 3:**
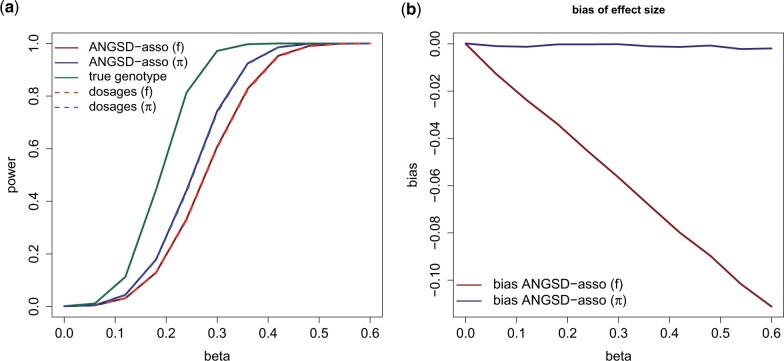
Simulation scenario 4 with varying genotype effect size (*β*). We have a structured population with the same admixture proportions and mean sequencing depth as in Figure  2C. There is an effect of ancestry of population 1 (*γ *= 1). We use a significance threshold of $10^{-3}$. The linear model is adjusted for ancestry. Each point is based on 10,000 simulations. (**A**) Statistical power to detect an association, using ANGSD-asso’s latent model and dosage model respectively with a sample frequency prior (f) and an individual allele frequency prior (*π*). (**B**) Bias of the estimated effect sizes.

In scenario 3, we simulate population structure and sequencing depth and phenotype correlation. As shown in Figure 2, using the sample allele frequency prior gives biased estimates of the effect size and leads to an increased false positive rate. The increased false positive rate is present even though we are adjusting for ancestry in the linear model, showing that this is not sufficient in this scenario. When using the individual allele frequency prior, we do not get biased estimates and have a false positive rate that is identical to using the true genotype. In scenario 4, we remove correlation between sequencing depth and phenotype and use a range of effect sizes for the genotype. Figure 3 shows that using the individual allele frequency prior has increased statistical power compared with the sample allele frequency prior. For example for an effect size of β=0.3 the power is 0.74 compared with 0.61. Furthermore, in this scenario, we see that using dosages has similar statistical power to using genotype probabilities. When using the sample allele frequency prior the effect sizes are underestimated. This is due to the fact that using the individual allele frequency better describes the expected genotype in a structured population.

In scenario 5, we remove population structure but reintroduce correlation between sequencing depth and phenotype and use a range of effect sizes for the genotype. Supplementary Figures S3b and S4b show that in this scenario there is slightly increased statistical power when using the full genotype probabilities compared with using dosages, and that our estimated effect size is less biased. In scenario 6, we have correlation between sequencing depth and phenotype, population structure and use a range of effect sizes for the genotype. Supplementary Figures S5b and S6b show that in this scenario there is increased statistical power when using the full genotype probabilities compared with using dosages, and less bias of the estimated effect size.

To evaluate the effect of estimating the admixture proportions from a limited number of genetic sites, we ran simulations with a varying number of sites used for estimating the admixture proportions. We observe a bias in effect size and a reduction in power when a small number (50 or 500) of sites is used. However, when the number of sites is increased (5000 or 50,000), there is very little reduction in power or bias (Supplementary Tables S3 and S4) compared with using the true admixture proportions.

### Comparison with dosages in large-scale studies

To further explore the performance difference with dosages, we simulated a large case-control study with 100,000 individuals. All individuals have low depth sequencing data but the cases and controls have different average sequencing depths.

In Table 2, we show that using full genotype probabilities has increased statistical power compared with when using genotype dosages. We have more power for small effect sizes, where we have a true positive rate that is almost 0.1 higher. We calculated the info measure for our dosages in cases and controls respectively, to make it comparable with haplotype imputation. To calculate the info measure, we used the ratio of observed variance of the dosages to the expected binomial variance at Hardy–Weinberg equilibrium, as used in the imputation software MACH (Scott et al. 2007). When genotypes are predicted with high certainty the info measure will be close to 1. We see that the info measure is lower in controls, where we have a lower average sequencing depth. Supplementary Figures S3, a and b and S4, a and b show that in scenario 5, with a quantitative trait, there is also increased statistical power and less bias when using full genotype probabilities. It also shows that in these simulations when keeping individuals with 0 reads, there is less bias but similar statistical power. In Table 3, we run the analysis from Table 2, but including individuals with 0 reads. In this scenario, the difference between using dosages and genotype probabilities has been almost erased. However, it is worth noticing that in this scenario, we lose statistical power compared with when we remove individuals with 0 reads. To further investigate these scenarios, we looked at the bias of the estimated effect sizes. Supplementary Figures S9 and S10 show for a relative risk (RR) of 1.14 that the latent model gives less biased estimates of the effect sizes compared with dosages.

**Table 2:** The statistical power for different simulated effect sizes or RRs of the genotype

	RR = 1	RR = 1.1	RR = 1.12	RR = 1.14	RR = 1.16
True genotype	0	0.587	0.868	0.978	0.999
Dosage	0	0.114	0.300	0.431	0.808
Genotype probabilities	0	0.163	0.388	0.659	0.862
*R* ^2^ cases/controls	0.91/ 0.85	0.91/ 0.85	0.91/ 0.84	0.90/ 0.84	0.90/ 0.84

The phenotype is simulated as a binary trait. We have performed 10,000 simulations for each effect size. The causal allele of the genetic variant has a frequency of 0.05 and the disease has a prevalence of 0.10 in the population. We use a significance threshold of $10^{-5}$. We have used the sample allele frequency prior as there is no population structure. We simulated 50,000 controls and cases respectively, with an average sequencing depth of 1*X* and 4*X*, respectively. We have removed individuals with 0 reads (0*X*). The *R*^2^ values denote how well the true genotype is predicted with this data and are calculated like the info measure used in the MACH imputation software (Scott *et al.* 2007).

**Table 3:** This table is similar to Table  2; it is the same scenario also, but where we include individuals with 0 reads.

	RR = 1	RR = 1.1	RR = 1.12	RR = 1.14	RR = 1.16
True genotype	0	0.813	0.974	0.999	1.000
Dosage	0	0.0914	0.262	0.523	0.772
Genotype probabilities	0	0.0974	0.273	0.538	0.783
*R* ^2^ cases/controls	0.91/ 0.77	0.90/ 0.75	0.90/ 0.75	0.90/ 0.75	0.90/ 0.75

Supplementary Figures S5, a and b and S6, a and b, show that in scenario 6, with a quantitative trait there is increased statistical power and less bias, when using genotype probabilities compared with dosages. Last, if we do scenario 6, but for a binary phenotype, we show increased statistical power and a smaller bias of the effect size, when using genotype probabilities compared with dosages as shown in Supplementary Figures S7, a and b and S8, a and b. And that in these simulations removing individuals with 0 reads, leads to increased statistical power and less bias.

### Comparison with SNPTEST

#### UK Biobank data

**Figure 4:**
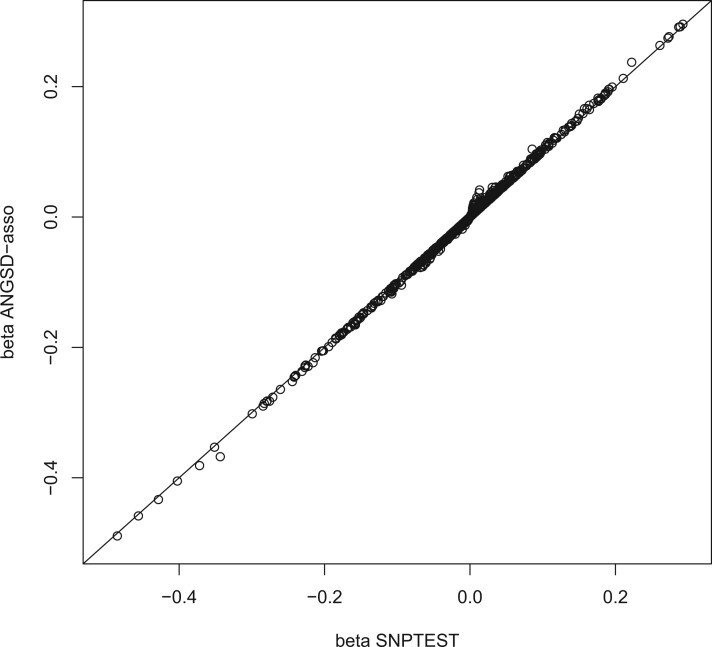
Estimated effect sizes from respectively SNPTEST and ANGSD-asso’s latent model of the overlapping 1596 genetic variants, between the two analyses. These estimates are based on the imputed data from the UK Biobank of a 50-kb region on chromosome 2 (219,675–219,725 kb). Both analyses have been adjusted for top 10 genetics PCs as provided by the UK Biobank data, age, gender, and BMI, the analyzed trait is waist–hip ratio from the UK Biobank data that has been inverse quantile transformed to a standard normal distribution.

SNPTEST (Marchini *et al.* 2007) also implements a latent model for performing association (using the option *-method em*) with genotype probabilities also using a GLM framework. We applied both methods to the imputed data of UK Biobank (Bycroft *et al.* 2018); more specifically, we chose a 50 kb region of chromosome 2 (219,675–219,725 kb), which has the genetic variant rs78058190. This variant has been found to be associated with waist–hip ratio in Kichaev *et al.* (2019) and the association is even stronger when adjusted for body mass index (BMI) (Pulit *et al.* 2019). It is an imputed variant in the UK Biobank data (info/*R*^2^ = 0.778797, minor allele frequency (MAF) = 0.049; Bycroft *et al.* 2018). We therefore ran both methods on the genotype probabilities from the imputation for this region, adjusting for gender, age, BMI, and top 10 genetic PCs (from UK Biobank), with waist–hip ratio as the phenotype but inverse quantile transformed to a standard normal distribution. ANGSD-asso’s latent model runs this analysis of 292,432 individuals and 1647 genetic variants in 82.13 min (17.71 min with 20 threads), SNPTEST runs this in 175.64 min (no multi-threaded option). The estimated effect sizes from each method are compared in Figure 4, showing that the estimated effect sizes are very similar. However, SNPTEST adjusts for covariates by first regressing out the covariates (plus an intercept) on the phenotype doing ordinary least squares. SNPTEST then subsequently uses the EM algorithm to obtain estimates of the intercept and genotype effect. This approach can lead to bias in the estimated effect size of the tested variant when adjusting with covariates that are both correlated with the phenotype and the tested variant (Freckleton 2002; Vansteelandt *et al.*, 2009). For example, when performing conditional analysis on other genetic variants, in order to determine if the tested variant might be the casual variant. We show using the UK Biobank data (Bycroft *et al.* 2018), that SNPTEST estimates lower effect sizes when adjusting for a covariate with high correlation to the focal variant (rs113414093) which is a consequence of regressing out the covariate prior to testing the variant (Table 4). When testing the three variants (rs1134114093, rs116204487, and rs148358468), but adjusting for rs78058190, we obtain similar results for ANGSD-asso and SNPTEST (Supplementary Tables S1 and S2).

**Table 4:** Waist–hip ratio association of rs78058190 in UK biobank

Covariates	SNPTEST *P*	SNPTEST *β*	ANGSD *P*	ANGSD *β*
Age, sex, BMI, and PC 1–10	8.02 × 10^–12^	0.030	7.62 × 10^−12^	0.030
Age, sex, BMI, and PC 1–10	0.00086	0.015	1.04 × 10^−6^	0.031427
rs113414093 (*R*^2^ 0.58)				
Age, sex, BMI, and PC 1–10	7.39 × 10^−6^	0.020	1.3 × 10^−6^	0.024
rs116204487 (*R*^2^ 0.20)				
Age, sex, BMI, and PC 1–10	1.21 × 10^−5^	0.019	7.14 × 10^−7^	0.024
rs148358468 (*R*^2^ 0.19)				

This table shows the *P*-value and estimated effect size (*β*), for the association of rs78058190 with waist–hip ratio (inverse quantile transformed to a standard normal distribution) when conditioning on genetic variants in linkage disequilibrium LD with rs78058190. The *R*^2^ values are shown in the table and are based on the LDproxy tool that is part of LDlink (Machiela and Chanock 2015). Results for rs113414093, rs116204487, and rs148358468 conditional on rs78058190 can be found in Supplementary Table S1 and S2.

#### Simulated data

SNPTEST can also be shown to be biased in simulations. We simulated a scenario with population structure that is both correlated with the phenotype and the genotype. Supplementary Figure S12 shows that in this scenario SNPTEST’s effect sizes are downward biased, whereas ANGSD-asso’s latent model has no bias, when using the individual allele frequency prior, and it also has increased statistical power compared with SNPTEST. We have used the most recent version of SNPTEST (v2.5.4-beta3). We ran all analyses with SNPTEST on the same data as ANGSD-asso, meaning disabling the option of transforming covariates and phenotype in SNPTEST.

As shown in Supplementary Figure S11 priming ANGSD-asso’s EM algorithm with the coefficients from regression on dosages drastically reduces the number of iterations needed for convergence of the EM algorithm.

**Figure 5:**
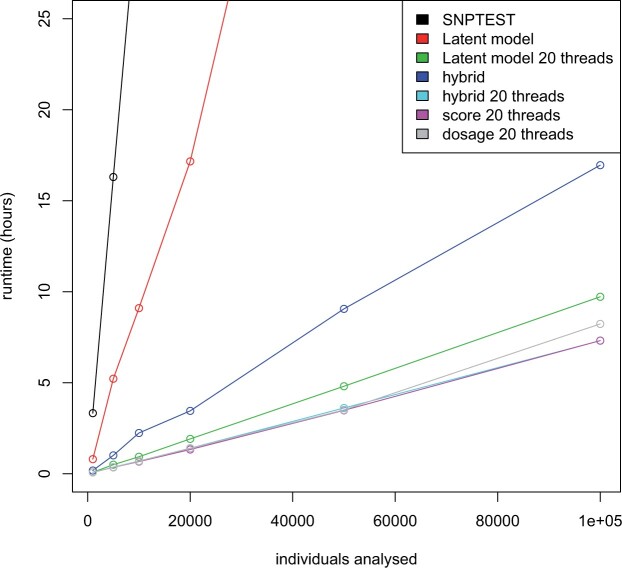
Running times for an analysis of a simulated binary trait with 442,769 genetic variants, varying the number of individuals (1000, 5000, 10,000, 20,000, 50,000, and 100,000), the model is run with two covariates (age and gender). The genetic data have an average depth of 1*X*. For each point the analysis was run three times and then used the mean running time. All analyses but the SNPTEST analysis were run in ANGSD.

We also compared ANGSD-asso’s latent model to SNPTEST in terms of computational speed and found that ANGSD-asso’s latent model is faster than SNPTEST, especially for binary data, as shown in Figure 5, and for quantitative data as shown in Supplementary Figure S13. ANGSD-asso’s latent model is capable of analyzing data sets of 100,000 individuals in <10 h. Our hybrid approach can handle the analyses in <17 h unthreaded. SNPTEST will take days to run the largest data set, when running a logistic model.

### Implementation of model

We have implemented ANGSD-asso’s latent model for taking genotype uncertainty into account when performing association studies. The advantage of this approach compared with the score test (Skotte et al. 2012), is that the effect size of the unobserved genotype is estimated. The effect size helps provide further insights into the relationship between genotype and phenotype. Furthermore, the estimated effect sizes also mean, we can make use of LD-score regression (Bulik-Sullivan et al. 2015). It is shown using UK Biobank data and simulations that ANGSD-asso’s latent model has increased statistical power and less bias compared with SNPTEST, when including covariates that are correlated with both phenotype and genotype in the model (Table 4 and Supplementary Figure S12). This is due to SNPTEST adjusting for covariates by first regressing them on the phenotype and then running the EM algorithm on the residuals. Including covariates in the linear model is a common way to deal with confounders in association studies. Also, it is shown that ANGSD-asso’s latent model is much faster than SNPTEST.

We have chosen to compare our method to SNPTEST as it is the only other method commonly used that takes genotype uncertainty into account. Additionally, almost all GWAS that use dosages are based on a standard GLM which will give identical results regardless of the method used. We therefore felt, it was not necessary to compare the dosage method with other software implementations. There are some exceptions to using standard GLMs. One of the exceptions is the use of dosages in linear mixed models which we have not explored in this study.

### Different priors in structured and homogeneous populations

We have shown how using the individual allele frequency prior, when estimating genotype probabilities, gives better statistical power to detect an association, when dealing with NGS data with population structure as shown in Figure 3. Also, it removes issues with an increased false positives rate when there is sequencing depth phenotype correlation as shown in Figure 2. This correlation might arise if the sequencing is not randomized, for example, if cases and controls are being sequenced at different times or places thereby creating a systematic bias, or if different cohorts have been sequenced at different places, or if the trait of interest is much more prevalent at one place *vs.* another. The scenarios from Table 1 are most likely to arise when dealing with nonmodel species where imputation cannot be done. This leads us to recommend using the individual allele frequency prior when performing association studies with NGS data in structured populations, where imputation is not possible. Whether it is better to base the individual allele frequency prior from clustering using NGSadmix (Skotte et al. 2013) or using PCA using PCAngsd (Meisner and Albrechtsen 2018) depends on the individuals in the data. If the sampled individuals are recent descendants of fairly discrete populations, such as most African Americans, then a clustering approach will give the most accurate allele frequencies while if the structure is more continuous such as many Latino Americans, then PCA might be a better approach.

### Comparison with dosages in large-scale studies

In Tables 2 and 3, we show through simulations increased statistical power when using genotype probabilities compared with dosages, with a larger gain in power for the scenario from Table 2. Since individuals with 0 reads are also removed from the true genotype in Table 2, there is higher statistical power for the true genotype in Table 3 as there are more individuals. In both instances, it is a case-control study with low depth sequencing data, where cases and controls have different average sequencing depths. A scenario like this, where there is better genotype information for some individuals, could also arise from haplotype imputation. As shown in Tables 2 and 3 with the info measure (*R*^2^) for controls and cases, where cases have more informative genetic data. This could happen if a certain population is not being represented in the reference panel used for imputation or if different reference panels or SNP-chips are used for cases and controls. A systematic difference in imputation quality is roughly equivalent to having a different average sequencing depth. However, at the same time, we have to state that our simulations and our analyses have shown us that in many instances dosages perform just as well. However, as mentioned there are certain circumstances where they do not perform as well. We have tried to explore these circumstances in this article and what the impact of them is.

Another conclusion, we can draw from our simulations is that when there is sequencing depth and phenotype correlation, our estimates of the effect size will be biased when we do not know the true frequency as shown in Supplementary Figures S4, S6, and S8. Furthermore, when there is sequencing depth and phenotype correlation it seems that keeping individuals with 0 reads makes a difference; however, this is due to how the simulations were performed and might not generalize to all scenarios.

With ANGSD-asso implemented in ANGSD, we have made it possible to perform large association studies with low depth sequencing data retaining maximal statistical power, and also estimating effect sizes. SNPTEST is too slow for the analysis of large-scale data sets. The speed-up of ANGSD-asso’s latent model compared with SNPTEST is due to priming for faster convergence of the EM algorithm and threaded analysis using the ANGSD (Korneliussen et al. 2014) framework. ANGSD-asso makes the analysis of large-scale data possible as done in Liu et al. (2018) (141,431 individuals) while retaining maximal statistical power. 

## Data availability

The three methods in ANGSD-asso for association analysis are implemented in the ANGSD framework. ANGSD can be downloaded from its github page: https://github.com/ANGSD/angsd.

The R-scripts used for generating the simulations are available from https://github.com/e-jorsboe/ANGSD-asso-scripts. Some of the simulations use population frequencies from (Lazaridis et al. 2014), this data set is available at https://reich.hms.harvard.edu/datasets. The imputed genetic data and phenotypes that are used, are available with the permission of the UK Biobank (https://www.ukbiobank.ac.uk). We conducted the research using the UK Biobank resource under an approved data request (ref: 32683).

Supplementary material is available at *G3* online.

## Supplementary Material

jkab385_Supplementary_Data

jkab385_Supplementary_Data2
