# Supplementary material for: Efficient approaches for large-scale GWAS with genotype uncertainty
Source: G3 (Bethesda). 2021 Dec 4;12(1):jkab385. doi: 10.1093/g3journal/jkab385 (PMC8727990; doi:10.1093/g3journal/jkab385)
Supplement: jkab385_Supplementary_Data [file jkab385_supplementary_data.pdf]

1 Supplementary Methods

1.1 `ANGSD-asso`'s hybrid model - for fast computation

The score test as described in Skotte et al. [2012] only has to estimate the parameters of the null model, where uncertainty on the variables do not have to be taken into account. It is therefore faster than our approach, where we both have to estimate the null and the alternative model. The idea behind the hybrid model is combining the speed of the score test with the desirable properties of `ANGSD-asso`'s latent model, where estimates of the effect size and standard error can be obtained. It works by first running the score test, and then if the site has a P-value below a certain threshold, we additionally run the slower `ANGSD-asso`'s latent model as well

$$p = \begin{cases} p_{score} < threshold & \Rightarrow \text{return } p_{EM} \\ p_{score} \geq threshold & \Rightarrow \text{return } p_{score}. \end{cases} \quad (1)$$

The threshold can be set by the user, the default value is 0.05.

1.2 `ANGSD-asso`'s dosage model

The expected genotype $E[G|\mathbf{x}]$ can easily be calculated from the genotype probabilities. This is an easy way to accommodate some of the genotype uncertainty and is therefore a method for trying to deal with genotype uncertainty in association studies

$$E[G|\mathbf{x}] = p(G = 1|\mathbf{x}) + 2p(G = 2|\mathbf{x}). \quad (2)$$

The genotype probability $p(G|\mathbf{x})$ can be calculated using the genotype likelihood $p(\mathbf{x}|G)$ and the frequency f of the genetic variant, for calculating the prior $p(G|f)$. This is done using Bayes' formula

$$p(G|\mathbf{x}, f) = \frac{p(\mathbf{x}|G)p(G|f)}{p(\mathbf{x})} = \frac{p(\mathbf{x}|G)p(G|f)}{\sum_{g\in 0,1,2} p(\mathbf{x}|G = g)p(G = g|f)}. \quad (3)$$

Here it is assumed that we have one homogeneous population where f describes the frequency of the genetic variant well across all individuals. Other priors can be used, for instance a prior based on the individual allele frequency (π) or haplotype frequencies. We perform standard ordinary least squares using $E[G|\mathbf{x}]$ as our explanatory variable

$$y_i = \sum_c \gamma_c z_{ic} + E[G_i|x_i]\beta + \epsilon_i. \quad (4)$$

1.3 Simulated sequencing data

Sequencing data was simulated by first choosing an average depth for a group of individuals and then sampling the specific depth assuming a Poisson distribution. For simplicity we assumed a constant error rate of 1 %, furthermore we

assumed that only two bases exist and sample the reads from these two alleles conditional on the simulated genotype and the error rate. For the run-times in Figure 5 and Supplementary Figure 13 and the effects of priming in Supplementary Figure 11, genetic data was simulated using frequencies from the Yoruba population from Lazaridis et al. [2014b] where the curated Human Origins data set was selected.

We chose 6 different simulation scenarios, as summarised in Table 1. In scenario 1 we evaluate the false positive rate when there is sequencing depth and phenotype correlation, under our null hypothesis of no effect of the genotype. In scenario 2 we examine the statistical power when simulating under our alternative hypothesis with no sequencing depth and phenotype correlation. Scenario 3 is similar to scenario 1 and 4 is similar to 2, but with the addition of population structure. Scenario 5 and 6 are similar to scenario 2 and 4 respectively, but with correlation between sequencing depth and phenotype correlation. The sequencing depth and phenotype correlation was simulated using a logistic function modelling the probability of being in the group with high average sequencing depth $p(D_i = \text{high})$. As this function maps the input into probabilities, also the steepness of the curve can conveniently be controlled with just one parameter.

$$p(D_i = \text{high}|\delta, y_i) = \frac{1}{1 + e^{(-\delta y_i)}} \tag{5}$$

The higher (in absolute value) δ is, to a larger degree the phenotype will correlate with being in the group of high or low average sequencing depth, meaning the lowest values of the phenotype will have the lowest probability of being in the high depth group whereas the highest values of the phenotype will have the highest probability of being in the high depth group (if $\delta > 0$ and vice versa if $\delta < 0$).

From the simulation data we estimate frequencies from the genotype likelihoods. For the admixed individuals we assume that the admixture proportions are known, we estimate the population frequencies using the approach from Skotte et al. [2013]. The sequencing depth and phenotype correlation is simulated as described in eq. 5.

1.4 Simulating estimated admixture proportions

We added simulations where we performed association analysis of the variant rs2951755. We used the curated Human Origins data set [Lazaridis et al., 2014a] with individuals from the Human Genetic Diversity Project (HGDP). In this data set rs2951755 has a frequency of 0.1 in French and 0.63 in Yoruba. We chose this variant because it has a high difference in population specific frequencies. We simulated genotypes for rs2951755 and between 50 to 50,000 variants based on frequencies from randomly sampled variants from the HGDP data. We simulated individuals as a mix between the French and Yoruba with similar admixture proportions to that of scenario 4 (see Figure 2) and with the same simulated effect size of 0.3 and an effect of ancestry of 1. The genotype likelihoods were then used to estimate the admixture proportions using NGSadmix

[Skotte et al., 2013]. The results are shown in Supplementary Table 3 & 4 when using both the known and estimated admixture proportions.

2 Simulating scenario 1 and 2

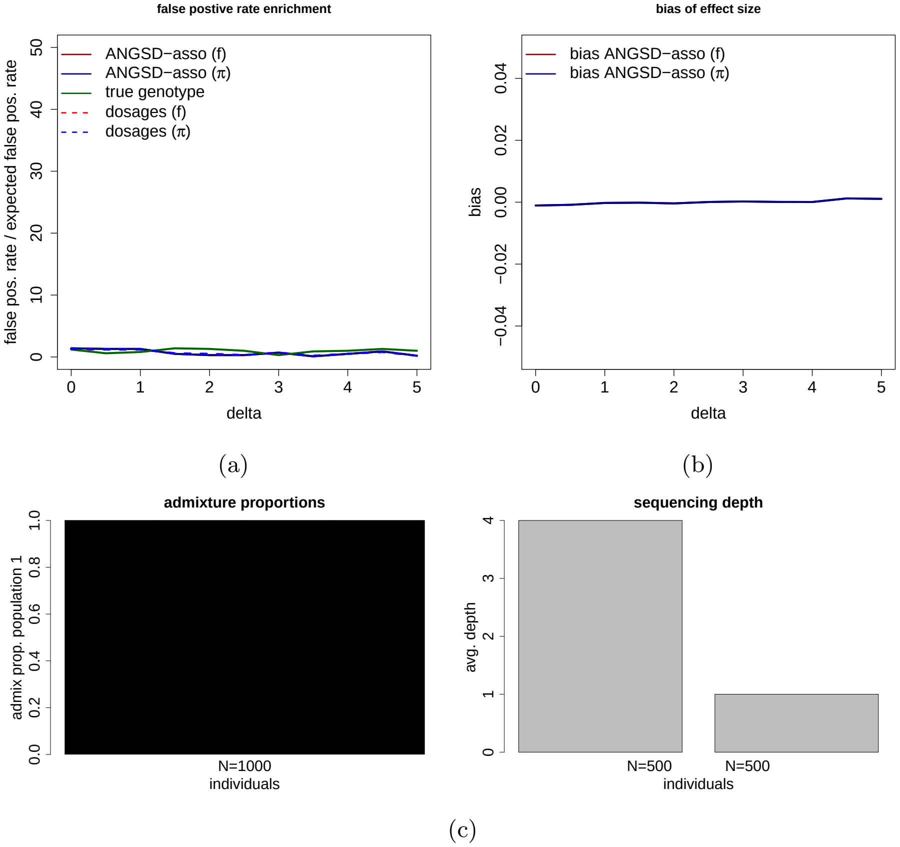

Figure 1: Simulation scenario 1 with varying the sequencing depth and phenotype correlation (δ) (eq. 5). We have a population with 1,000 individuals without population structure or effect of genotype. We use a significance threshold of 10^{-3}. Each point is the mean value from 10,000 simulations. **(a)**: False positive rate divided by the expected false positive rate (10^{-3}) as a function of the sequencing depth phenotype correlation, using `ANGSD-asso`'s latent model and dosage model respectively with a sample frequency prior (f) and an individual allele frequency prior (π). **(b)** Bias of the estimated effect sizes. **(c)** The simulated admixture proportions and the mean sequencing depth for the simulated individuals.

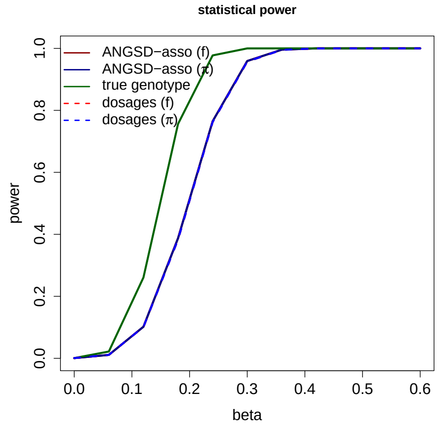

(a)

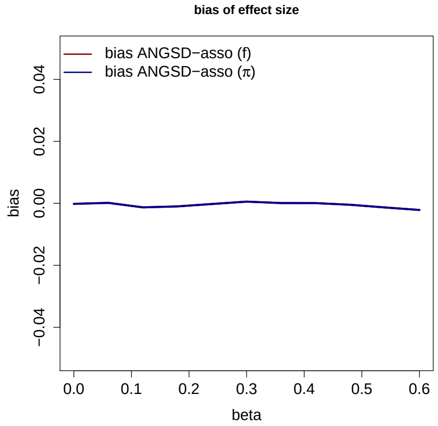

(b)

Figure 2: Simulation scenario 2 with varying the genotype effect size (β) for a population without structure. We have the same mean sequencing depth as in Supplementary Figure 1 (c). The phenotype is simulated as a quantitative trait, with different effect sizes of the genotype, for each tested effect size it is the mean value from 10,000 simulations. **(a)**: Statistical power to detect a true association with a significance threshold of (10^{-3}), using `ANGSD-asso`'s latent model and dosage model respectively with a sample frequency prior (f) and an individual allele frequency prior (π). **(b)**: Bias of the estimated effect sizes.

3 Sequencing depth phenotype correlation with a quantitative phenotype

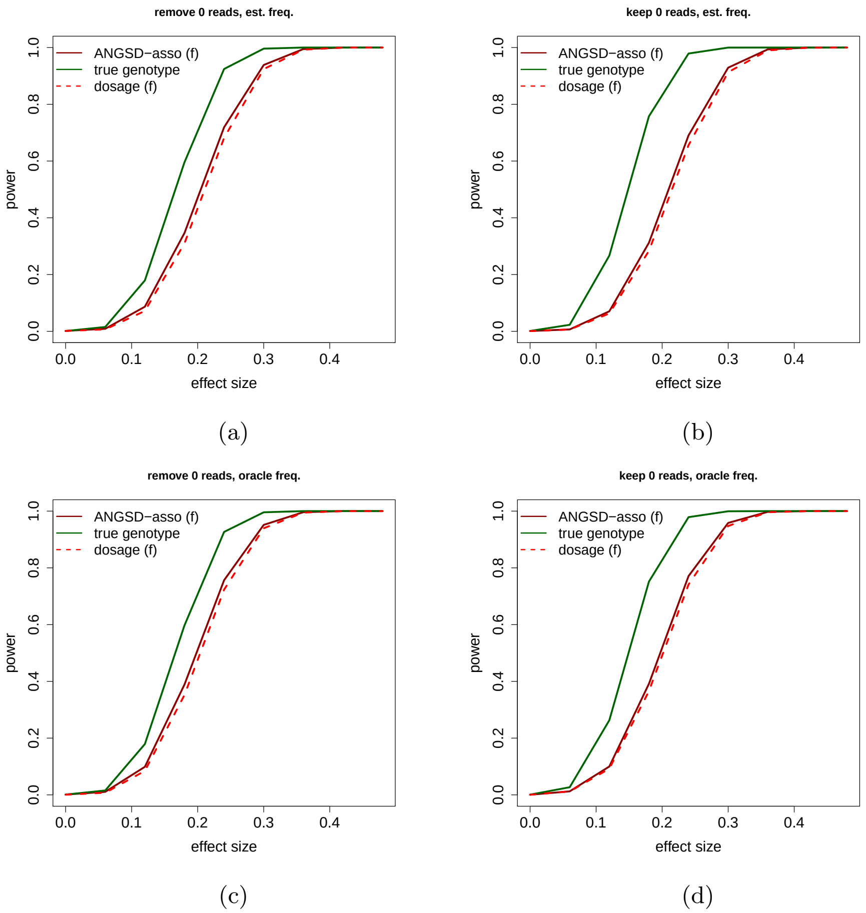

Figure 3: Simulation scenario 5 with varying the effect size of the genotype (β) and with sequencing depth phenotype correlation ($\delta = 5$, eq. 5). The phenotype is simulated as a quantitative trait, with different effect sizes of the genotype, for each tested effect size it is the power calculated from 10,000 simulations. **(a)**: Statistical power to detect a true association with a significance threshold of (10^{-3}), using `ANGSD-asso`'s latent model and dosage model respectively with a sample frequency prior (f), removing individuals with 0 reads and estimating f from the genotype likelihoods. **(b)**: Like (a) but keeping individuals with 0 reads. **(c)**: Like (a) but knowing the simulated f. **(d)**: Like (a) but keeping individuals with 0 reads and knowing the simulated f.

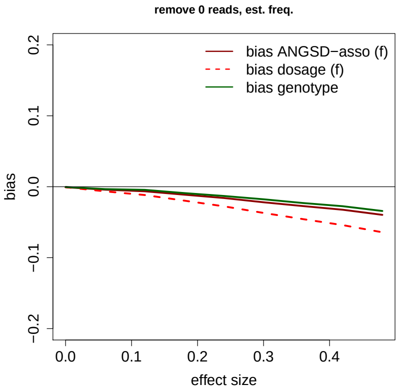

(a)

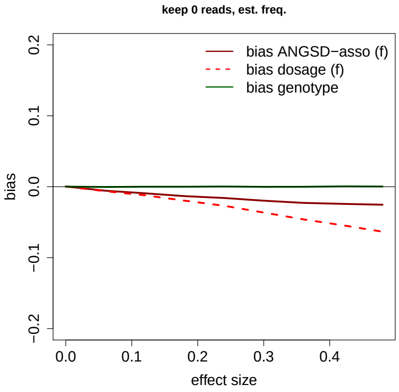

(b)

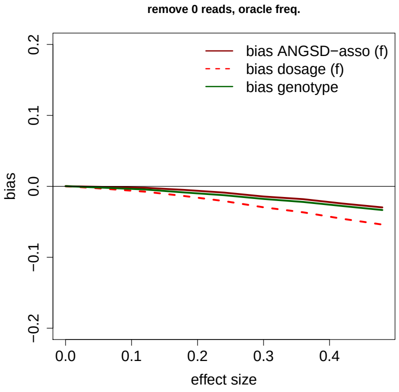

(c)

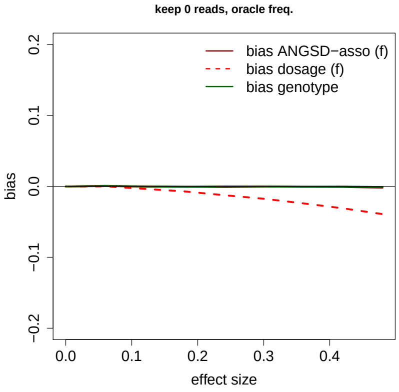

(d)

Figure 4: Like Supplementary Figure 3, but showing the bias of the estimated effect size.

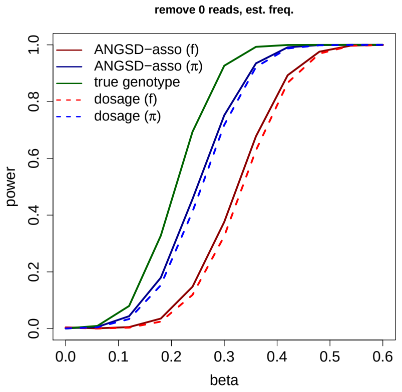

(a)

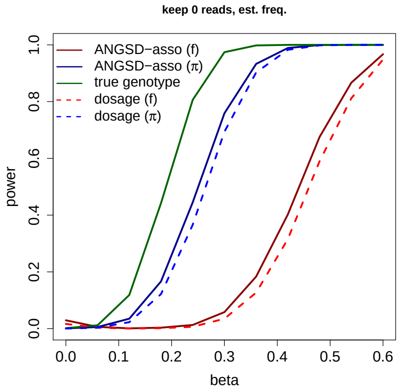

(b)

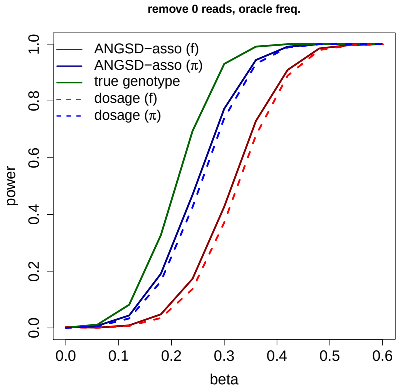

(c)

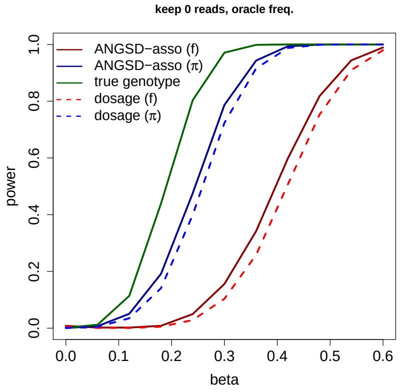

(d)

Figure 5: Simulation scenario 6 with varying genotype effect size (β), sequencing depth phenotype correlation (δ) is fixed at a value of 5 (eq. 5). There is an effect of ancestry of population 1 ($\gamma = 1$). The phenotype is simulated as a quantitative trait, for each tested effect size it is the mean power from 10,000 simulations. **(a)**: We show the statistical power to detect a true association with a significance threshold of (10^{-3}), using `ANGSD-asso`'s latent model and dosage model respectively with a sample frequency prior (f) and an individual allele frequency prior (π). **(b)**: Like (a) but keeping individuals with 0 reads. **(c)**: Like (a) but knowing the simulated f and π. **(d)**: Like (a) but keeping individuals with 0 reads and knowing the simulated f and π.

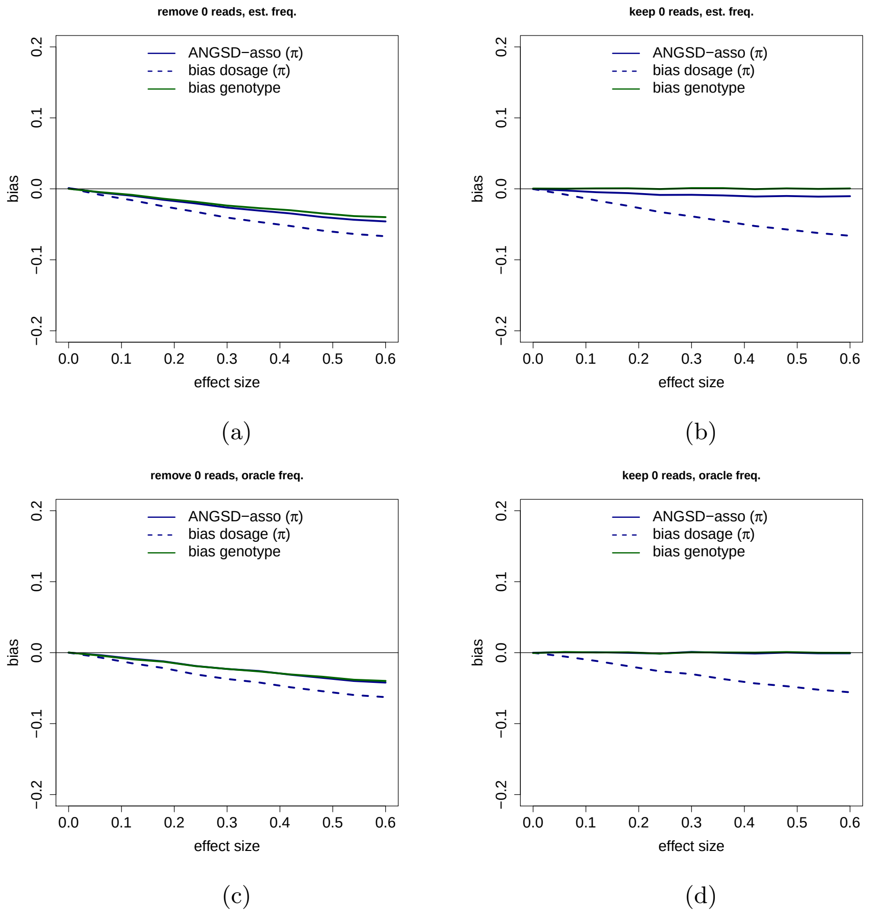

Figure 6: Like Supplementary Figure 5, but showing the bias of the estimated effect size.

4 Sequencing depth phenotype correlation with a binary phenotype and population structure

The binary phenotype is simulated like this:

$$p(y = 1) = \frac{1}{1 + e^{-(\beta \cdot g + \gamma \cdot q)}} \tag{6}$$

Depth phenotype correlation was simulated like this:

$$p(D_{high}|y = 1) = \frac{1}{1 + e^{-\delta}} \tag{7}$$

$$p(D_{high}|y = 0) = 1 - \frac{1}{1 + e^{-\delta}} \tag{8}$$

The higher δ is the more probable cases will be in the high depth category.

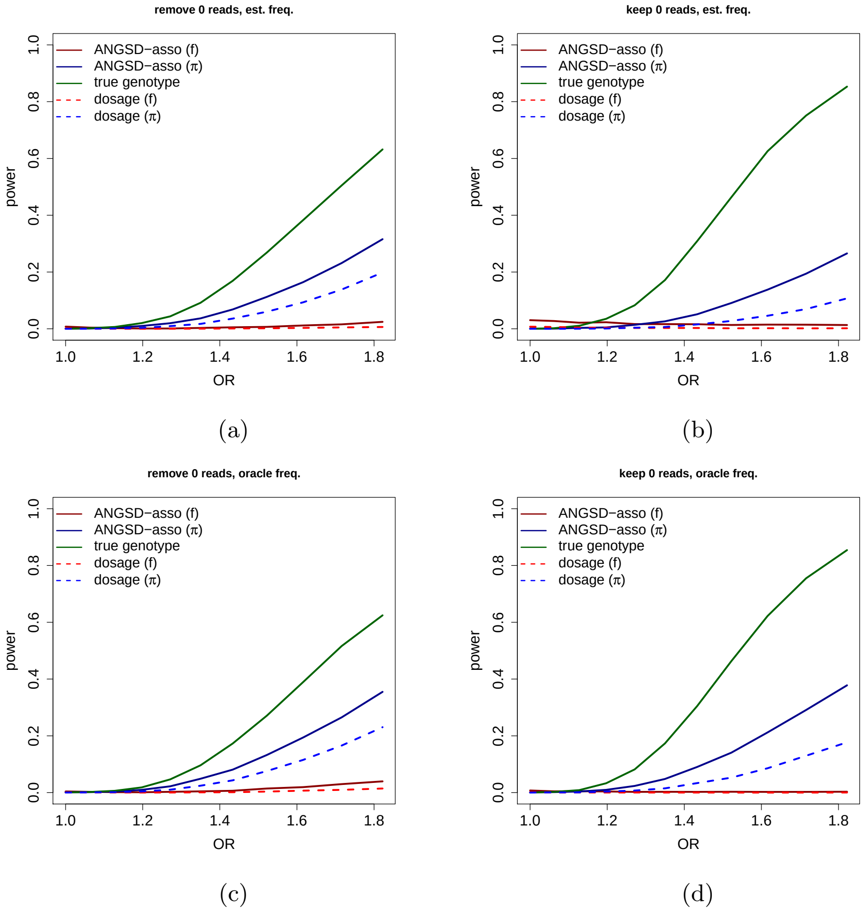

Figure 7: Simulations like scenario 6 but for a binary phenotype instead of a quantitative as in supplementary figure 5.

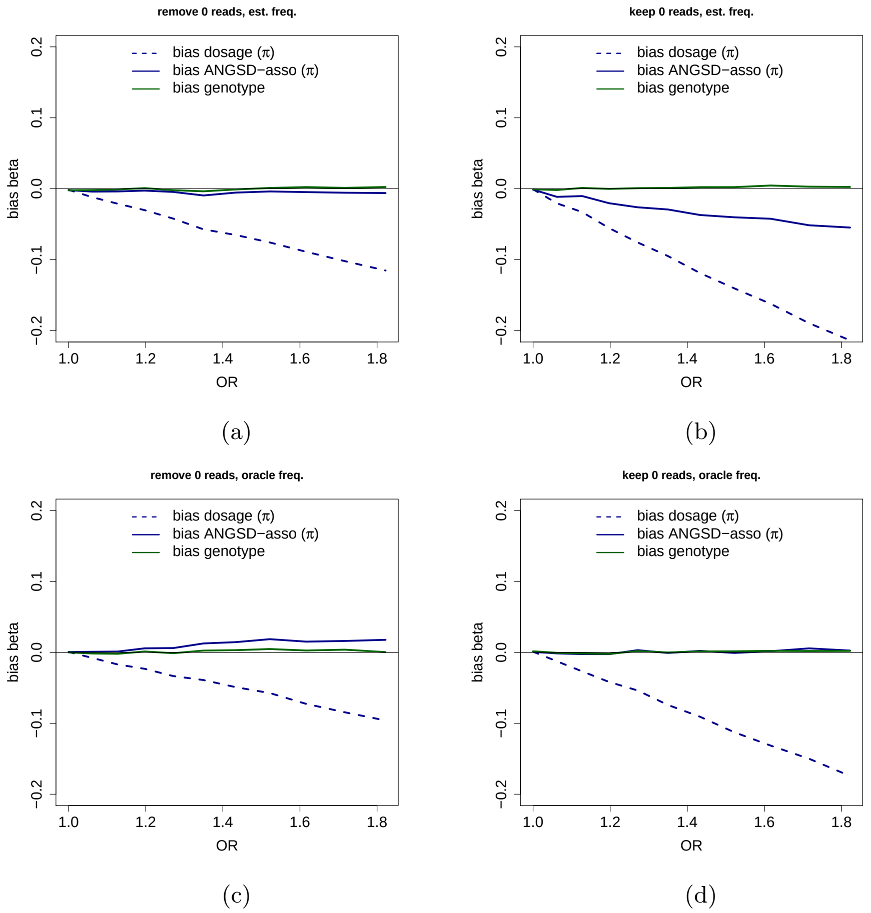

Figure 8: Like Supplementary Figure 7, showing the bias of the estimated effect size (β) not the odds ratio.

5 Sequencing depth phenotype correlation with a binary phenotype

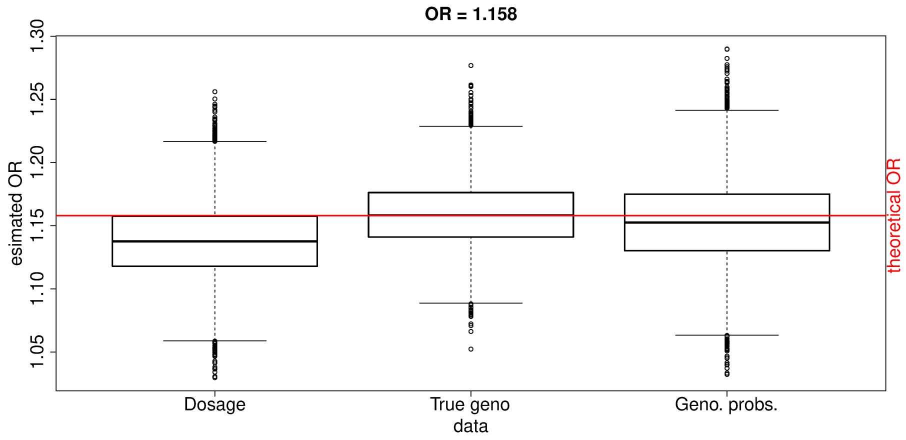

Figure 9: Estimated odds ratio (OR) from all 3 methods used in Table 2, in order to show the bias of the estimated effect size. The estimated effect sizes were simulated using a relative risk (RR) of 1.14. For showing the bias of the estimated effect size, the OR is used, as this can be obtained from the logistic regression. A RR of 1.14 with a disease prevalence of 0.1 is equivalent to an OR of 1.158. The formula for converting OR to RR from Zhang and Kai [1998] was used.

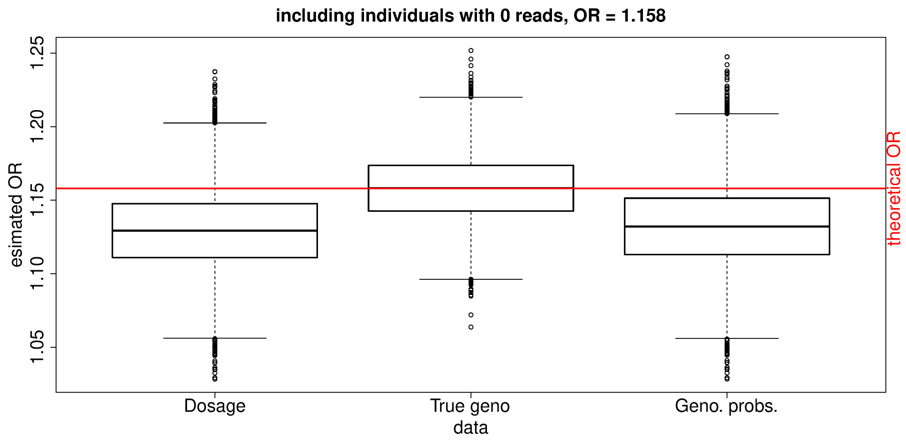

Figure 10: Like Supplementary Figure 9, but keeping individuals with 0 reads, like in Table 3.

5.1 Priming coefficients for faster convergence

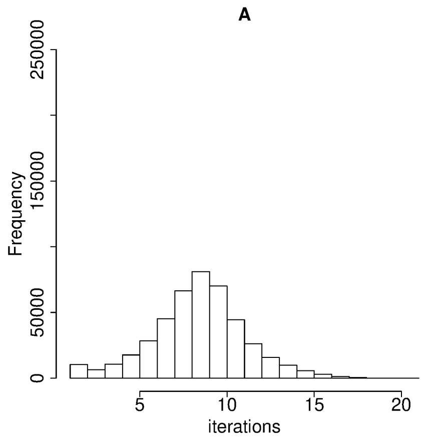

(a)

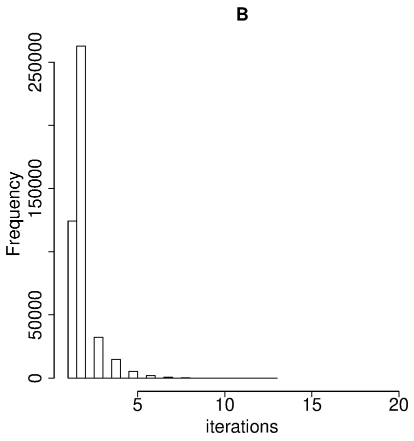

(b)

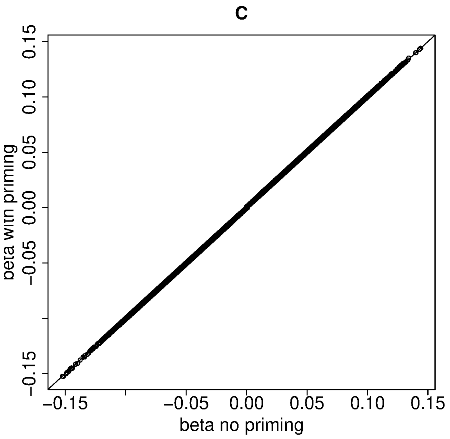

(c)

Figure 11: **(a)**: Iterations for the analysis of 442,769 sites for 5,000 individuals with a simulated quantitative trait. Sequencing depth is on average $1X$ for the individuals when not priming coefficients. **(b)**: Same analysis as **(a)** but when priming coefficients. **(c)**: Effect sizes for `ANGSD-asso`'s latent model when not using priming and when using priming.

5.2 Genotype likelihoods from NGS data

Next generation sequencing (NGS) produces reads with the observed nucleotide bases. These reads are then aligned to a reference genome, and each position will be covered by a certain number of reads, the more reads covering a position the more certainty there is of the true genotype of that position. The number of reads covering a position is called the sequencing depth. Each base in a sequenced read will have a quality score denoting the certainty of the called nucleotide base. Genotype likelihoods (GLs) is the likelihood of observing the sequence data x_j given an unknown genotype G, meaning $p(x_j|G)$ for a given position in the genome j. The sequence data x_j will consist of R observed bases $x_j = (b_1, b_2, ..., b_R)$.

The GL $p(x_j|G)$ is calculated using the quality scores of x_j, one method for doing so is by assuming the R observed bases are independent given our genotype G

$$p(X_j|G) \propto \prod_{r=1}^{R} p(b_r|G) = \prod_{r=1}^{R} p(b_r|A_1, A_2) =$$

Where A_1 and A_2 are the two alleles of the genotype G, summing over the alleles A and rewriting the likelihood

$$\prod_{r=1}^{R} \sum_{A \in A_1, A_2} p(b_r|A)p(A) =$$

And since $p(A)$ or the probability of observing one of the two alleles must be one half.

$$\prod_{r=1}^{R} \left(\frac{1}{2} p(b_r|A_1) + \frac{1}{2} p(b_r|A_2) \right).$$

The probability of a base b given an allele A relates to the error rate ϵ.

$$p(b|B) = \begin{cases} \frac{\epsilon}{3}, & b \neq A \\ 1 - \epsilon, & b = A \end{cases}$$

Here R is the depth at site j, b_r is the observed rth base, and ϵ is the probability of an error as calculated from the quality score of b_r.

This is the approach used in the GATK framework McKenna et al. [2010], other approaches where the quality score is not used directly exist, for example SAMtools Li et al. [2009].

The likelihood can be used to calculate the posterior probability of the genotype G or the genotype probability, using a genotype prior, $p(G)$ and Bayes Theorem, like in eq. 3.

6 Poisson distribution written as an exponential family

For the Poisson distribution we have that the probability of our phenotype y_i of the ith individuals with value k can be written like this

$$p(y_i = k|G_i, \mathbf{z_i}) = \frac{\lambda_i^k e^{-\lambda_i}}{k!}. \tag{9}$$

Where our linear predictor for the Poisson regression is

$$\eta_i = \log E[y_i] = \log \lambda_i. \tag{10}$$

We can write up $\exp(\log(p(y_i = k|G_i, \mathbf{z_i}))$

$$\exp\left(\log\left(\frac{\lambda_i^{y_i} e^{-\lambda_i}}{y_i!}\right)\right) = \exp(\log \lambda_i^{y_i} e^{-\lambda_i} - \log(y_i!)) = \tag{11}$$

$$\exp(y_i \log \lambda_i + \log e^{-\lambda_i} - \log(y_i!)) = \exp(y_i \log \lambda_i - \lambda_i - \log(y_i!)). \tag{12}$$

Then we can write it as an exponential family using notation from Dobson and Barnett [2008]

$$\exp\left(\frac{y_i \eta_i - b(\eta_i)}{a(\phi)} + c(y_i, \phi)\right). \tag{13}$$

Giving us $a(\phi) = 1, b(\eta_i) = \lambda_i = \exp(\eta_i)$ and $c(y_i, \phi) = -\log(y!)$.

7 EM algorithm in `ANGSD-asso`'s latent model

Starting from eq. 4 in the main text where we have

$$\sum_i^N \log\left(\sum_{g\in\{0,1,2\}} p(y_i|G_i = g, \mathbf{z_i}, \theta) p(G_i = g|x_i)\right). \tag{14}$$

The term $p(y_i|G_i, \mathbf{z_i}, \theta)$ we base on a linear regression model. We assume that given the genotype G, covariates Z and parameters θ the phenotype follow a normal distribution with a mean given by

$$\eta_i = \alpha + \beta G_i + \sum_c \gamma_c z_{ic}. \tag{15}$$

We can then apply the EM algorithm for maximising the likelihood. First we do the E-step, the term $p(y_i|G_i, z_i, \theta)$ is the only one that depends on θ, this is

equivalent to maxisming

$$E_{G|\mathbf{y},\mathbf{x},Z,\theta}[\log p(\mathbf{y}|G_i,Z,\theta)] = E_{G|\mathbf{y},\mathbf{x},Z,\theta}[\log \prod_i^N p(y_i|G_i,\mathbf{z_i},\theta)] \tag{16}$$

$$= \sum_i^N E_{G|y_i,x_i,\mathbf{z_i},\theta}[\log p(y_i|G_i,\mathbf{z_i},\theta)]. \tag{17}$$

And then the M-step is differentiating this as in Lake et al. [2003]

$$\frac{\partial}{\partial\beta}\sum_i^N E_{G|y_i,x_i,\mathbf{z_i},\theta}[\log p(y_i|G_i,\mathbf{z_i},\theta)] \tag{18}$$

$$= \sum_i^N E_{G|y_i,x_i,\mathbf{z_i},\theta}\left[\frac{\partial}{\partial\beta}\log p(y_i|G_i,\mathbf{z_i},\theta)\right] \tag{19}$$

$$= \sum_i^N E_{G|y_i,x_i,\mathbf{z_i},\theta}\left[\frac{\partial\eta_i}{\partial\beta}\frac{\partial}{\partial\eta_i}\log p(y_i|G_i,\mathbf{z_i},\theta)\right] \tag{20}$$

Using the rule of differentiating a sum going from (18) to (19). Using the chain rule going from (19) to (20). ($\frac{dz}{dx} = \frac{dz}{dy}\frac{dy}{dx}$)

The term $p(y_i|G_i,\mathbf{z_i},\theta)$ can be written as an exponential family

$$p(y_i|G_i,\mathbf{z_i},\theta) = \frac{1}{\sqrt{2\pi\sigma^2}}\exp\frac{-(y-\eta_i)^2}{2\sigma^2}$$

$$= \exp\left(\frac{y_i\eta_i - \eta_i^2/2}{\sigma^2} - y_i^2/2\sigma^2 - \log(2\pi\sigma^2)/2\right) = \exp\left(\frac{y_i\eta_i - b(\eta_i)}{a(\sigma)} + c(y_i,\sigma)\right).$$

Where $a(\sigma) = \sigma^2$, $b(\eta_i) = \eta_i^2/2$ and $c(y_i,\sigma) = -y_i^2/2\sigma^2 - \log(2\pi\sigma^2)/2$. For a logistic regression we have $a(\sigma) = 1$, $b(\eta_i) = \log(1+e^{\eta_i})$ and $c(y_i,\sigma) = 0$. And for a Poisson regression we have $a(\phi) = 1, b(\eta_i) = \lambda_i = \exp(\eta_i)$ and $c(y_i,\phi) = -\log(y!)$.

In the case of $b(\eta_i) = \eta_i^2/2$ and $a(\sigma) = \sigma^2$ (where $b'(\eta_i) = \eta_i$), we can de-

rive the following.

$$\sum_i^N E_{G|y_i,x_i,\mathbf{z_i},\theta}\left[\frac{\partial\eta_i}{\partial\beta}\frac{\partial}{\partial\eta_i}\frac{y_i\eta_i - b(\eta_i)}{a(\sigma)} + c(y_i,\sigma)\right] \tag{21}$$

$$= \sum_i^N E_{G|y_i,x_i,\mathbf{z_i},\theta}\left[\frac{\partial\eta_i}{\partial\beta}\frac{y_i - b'(\eta_i)}{a(\sigma)}\right] \tag{22}$$

$$= \sum_i^N E_{G|y_i,x_i,\mathbf{z_i},\theta}\left[G_i\frac{y_i-\eta_i}{\sigma^2}\right] \tag{23}$$

$$= \sum_i^N \sum_{g\in\{0,1,2\}}\left(G_i\frac{y_i-\eta_i}{\sigma^2}\right)p(G_i = g|y_i,x_i,\mathbf{z_i},\theta). \tag{24}$$

In going from (23) to (24) we are taking the expectation across all values of G. For the other variables (α, γ) in (15)

$$\sum_i^N \sum_{g\in\{0,1,2\}}\left(z_i\frac{y_i-\eta_i}{\sigma^2}\right)p(G_i = g|y_i,x_i,\mathbf{z_i},\theta). \tag{25}$$

Where for α we will just replace z_i with 1.

We recognise (24) and (25) as the score functions of a weighted regression, with regards to the respective terms (α, β, γ) [Dutang, 2017]. Each individual i, contributes one observation per possible genotype G the weights are given by $p(G_i|y_i,x_i,\mathbf{z_i},\theta)$ which is the probability of a genotype G given the phenotype y, covariates $\mathbf{z}$ and parameters θ. This is maximised by doing weighted least squares, where the parameters θ are chosen to maximise the likelihood.

The term $p(G_i|y_i,x_i,\mathbf{z_i},\theta)$ can be estimated using Bayes' theorem again making use of the assumption $p(G_i|x_i,\mathbf{z_i},\theta) = p(G_i|x_i)$, and that we can ignore the sequence data when we have the genotype $p(y_i|G_i,x_i,\mathbf{z_i},\theta) = p(y_i|G_i,\mathbf{z_i},\theta)$ this yields

$$p(G_i|y_i,x_i,\mathbf{z_i},\theta) = \frac{p(y_i|G_i,\mathbf{z_i},\theta)p(G_i|x_i)}{\sum_{g\in\{0,1,2\}} p(y_i|G_i = g,\mathbf{z_i},\theta)p(G_i = g|x_i)}. \tag{26}$$

7.1 Optimisation strategy for a normal distributed phenotype

First an initial guess of the standard deviation is calculated from the phenotype, using the sample standard deviation

$$s = sd[y] \tag{27}$$

Then linear regression is done with the full model, using the dosages calculated form the genotype probabilities, to estimate the an initial guess of the coefficients

for ANGSD-asso's latent model for faster convergence. This is referred to in this article as priming the coefficients.

Regression weights are then calculated according to (26) and weighted least squares is done using the parameters and weights from the last iteration of the EM algorithm. Each individual has three entries in the design matrix $(\mathbf{G}, Z)$, where $\mathbf{G}$ is a vector with each of the three possible genotypes for each individual, each weighted by $p(G_i|y_i, x_i, \mathbf{z_i}, \theta)$ as estimated for that individual and for that genotype. Then s is updated by using the weighted sum of squared residuals, from the weighted least squares with $n - o$ degrees of freedom. Where n is the number of individuals and o is the number of coefficients in the linear model as described in (15). The term $p(y_i|G_i, \mathbf{z_i}, \theta)$ can be calculated using a normal distribution with the following parameters, where η_i is from (15).

$$p(y_i|G_i, \mathbf{z_i}, \theta) = \mathcal{N}(\eta_i, s^2). \tag{28}$$

8 SNPTEST bias with simulated data

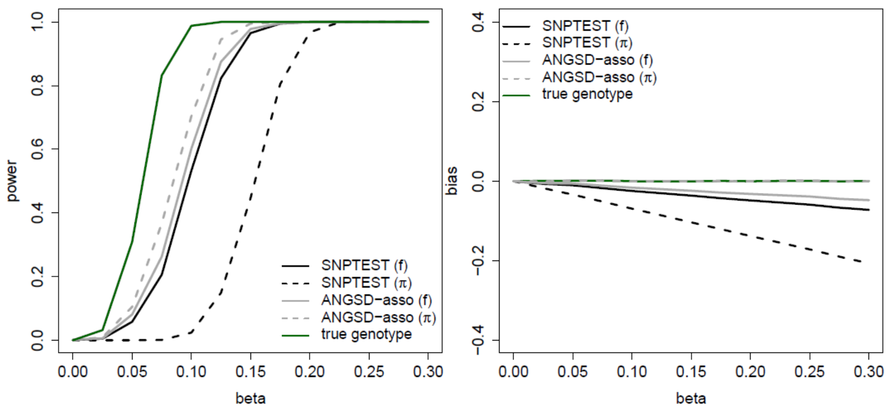

(a) (b)

Figure 12: This data is simulated with 10,000 individuals with an average depth of $0.1, 1, 10, 20X$, 2,500 individuals each. Varying the effect size of the genotype (β). There is an effect of ancestry of population 1 ($\gamma = 0.5$), the admixture proportions for population 1 are for the same 2,500 individuals each (Q_1=0.15, 0.4, 0.95, 1). We use a significance threshold of 10^{-3}. The linear models are adjusted for ancestry, SNPTEST was run without transforming the phenotype or covariates to make it as comparable to ANGSD-asso's latent model as possible. Each point is based on 1,000 simulations. **(a)**: We show the statistical power to detect a true association using ANGSD-asso's latent model and SNPTEST's latent model respectively with a sample frequency prior (f) and an individual allele frequency prior (π). **(b)**: We show the bias of our estimated effect size.

9 Running times for a quantitative trait

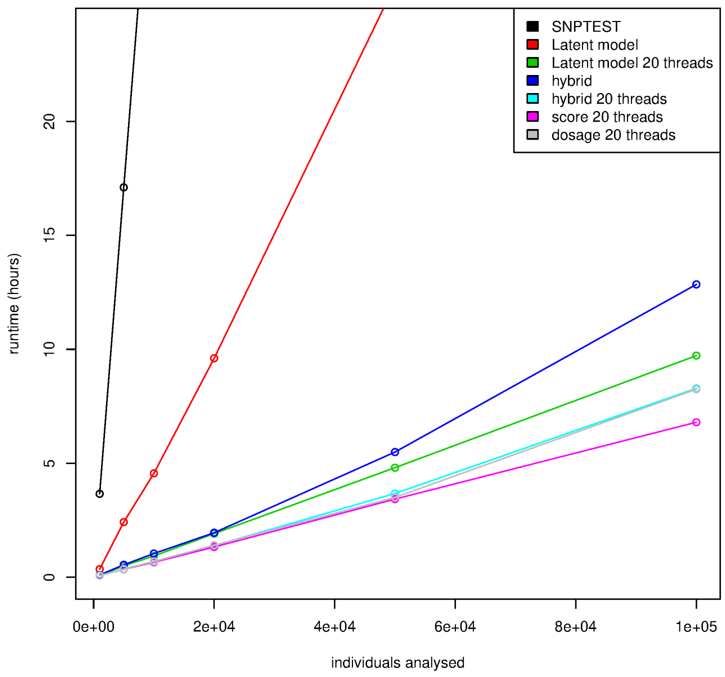

Figure 13: Running times for an analysis of a simulated quantitative trait with 442,769 genetic variants, varying the number of individuals (1,000, 5,000, 10,000, 20,000, 50,000 and 100,000), the model is run with 2 covariates (age and gender). The genetic data has an average depth of $1X$. For each point we have run the analysis 3 times and then used the mean running time. All runs but the SNPTEST analysis is run in `ANGSD`.

10 UK Biobank data adjusted for rs78058190

analysis:	ANGSD P	ANGSD β	ANGSD P (adj.)	ANGSD (adj.) β
age, sex, BMI, PC 1-10 rs113414093 (R^2 0.58)	$1.56 \cdot 10^{-6}$	0.022	0.79	-0.0017
age, sex, BMI, PC 1-10 rs116204487 (R^2 0.20)	$2.91 \cdot 10^{-8}$	0.022	0.0035	0.013
age, sex, BMI, PC 1-10 rs148358468 (R^2 0.19)	$6.49 \cdot 10^{-9}$	0.023	0.0013	0.014

Table 1: This Table shows the P-value and estimated effect size (β), for the association of respectively rs113414093, rs116204487 and rs148358468 with waist-hip ratio (inverse quantile transformed to a standard normal distribution) run with (adj.) and without conditioning on the genetic variant rs78058190, the R^2 values are shown in the table and are based on the LDproxy tool that is part of LDlink [Machiela and Chanock, 2015].

analysis:	SNPTEST P	SNPTEST β	SNPTEST P (adj.)	SNPTEST (adj.) β
age, sex, BMI, PC 1-10 rs113414093 (R^2 0.58)	$1.60 \cdot 10^{-6}$	0.022	0.86	-0.00078
age, sex, BMI, PC 1-10 rs116204487 (R^2 0.20)	$3.03 \cdot 10^{-8}$	0.022	0.0083	0.010
age, sex, BMI, PC 1-10 rs148358468 (R^2 0.19)	$6.79 \cdot 10^{-9}$	0.023	0.0038	0.011

Table 2: This Table shows the same as Supplementary Table 1, but using SNPTEST.

11 Power and bias when using estimated admixture proportions

Sites used for estimation of q	$bias_{estimatedQ}$	$bias_{knownQ}$
50	-0.10	-0.00073
500	-0.015	-0.00071
5,000	-0.0029	-0.00070
50,000	-0.0016	-0.000655

Table 3: This table shows the bias for doing association analysis respectively using known admixture proportions or using estimated admixture proportions. It is simulated like in Figure 2 with 1,000 individuals 1,000 runs for each number of sites and an effect size of the genotype of 0.3, and $\gamma = 1$.

Sites used for estimation of q	$power_{geno}$	$power_{estimatedQ}$	$power_{knownQ}$
50	0.985	0.301	0.833
500	0.985	0.769	0.833
5,000	0.985	0.820	0.833
50,000	0.985	0.830	0.833

Table 4: This table is like Supplementary Table 3, but showing the statistical power, using a threshold of 0.001.

References

Annette J Dobson and Adrian G Barnett. *An introduction to generalized linear models*. Chapman and Hall/CRC, 2008.

Christophe. Dutang. Some explanations about the iwls algorithm to fit generalized linear models. 2017.

S. L. Lake, H. Lyon, K. Tantisira, E. K. Silverman, S. T. Weiss, N. M. Laird, and D. J. Schaid. Estimation and tests of haplotype-environment interaction when linkage phase is ambiguous. *Hum. Hered.*, 55(1):56–65, 2003.

Iosif Lazaridis, Nick Patterson, Alissa Mittnik, Gabriel Renaud, and et al. Ancient human genomes suggest three ancestral populations for present-day europeans. *Nature*, 513(7518):409–413, Sep 2014a.

Iosif Lazaridis, Nick Patterson, Alissa Mittnik, Gabriel Renaud, Swapan Mallick, Karola Kirsanow, Peter H Sudmant, Joshua G Schraiber, Sergi Castellano, Mark Lipson, et al. Ancient human genomes suggest three ancestral populations for present-day europeans. *Nature*, 513(7518):409–413, 2014b.

Heng Li, Bob Handsaker, Alec Wysoker, Tim Fennell, Jue Ruan, Nils Homer, Gabor Marth, Goncalo Abecasis, and Richard Durbin. The sequence alignment/map format and samtools. *Bioinformatics*, 25(16):2078–2079, 2009.

Mitchell J Machiela and Stephen J Chanock. Ldlink: a web-based application for exploring population-specific haplotype structure and linking correlated alleles of possible functional variants. *Bioinformatics*, 31(21):3555–3557, 2015.

Aaron McKenna, Matthew Hanna, Eric Banks, Andrey Sivachenko, Kristian Cibulskis, Andrew Kernytsky, Kiran Garimella, David Altshuler, Stacey Gabriel, Mark Daly, et al. The genome analysis toolkit: a mapreduce framework for analyzing next-generation dna sequencing data. *Genome research*, 20(9):1297–1303, 2010.

L. Skotte, T. S. Korneliussen, and A. Albrechtsen. Association testing for next-generation sequencing data using score statistics. *Genet. Epidemiol.*, 36(5): 430–437, Jul 2012.

Line Skotte, Thorfinn Sand Korneliussen, and Anders Albrechtsen. Estimating individual admixture proportions from next generation sequencing data. *Genetics*, 195(3):693–702, Nov 2013. doi: 10.1534/genetics.113.154138.

Jun Zhang and F Yu Kai. What's the relative risk?: A method of correcting the odds ratio in cohort studies of common outcomes. *Jama*, 280(19):1690–1691, 1998.
